# Association between tumor size and prognosis in bladder cancer: novel classifications and insights from a SEER database analysis

**DOI:** 10.3389/fsurg.2024.1489832

**Published:** 2024-11-25

**Authors:** Yige Jia, Kan Wu, Xiang Li

**Affiliations:** Department of Urology, Institute of Urology, West China Hospital, Sichuan University, Sichuan, Chengdu, China

**Keywords:** bladder cancer, tumor size, prognosis, SEER database, restrict cubic spline

## Abstract

**Objective:**

Although tumor size is an essential oncologic feature, it is often underutilized in diagnosing and treating bladder cancer (BC). This study investigates the relationship between tumor size and BC prognosis, aiming to enhance clinical applications.

**Methods:**

BC patients were identified from the Surveillance, Epidemiology, and End Results (SEER) database (2004–2015). Cox proportional hazard models were conducted to identify prognostic factors, and restricted cubic splines (RCS) were used to assess the relationship between tumor size and survival outcomes. The Kaplan-Meier method and multivariate COX models were utilized to estimate the effect of the classification scheme.

**Results:**

A total of 69,478 patients with BC were evaluated from the SEER database. Larger tumor size, recent diagnosis, older age, high pathologic grade, variant histology, advanced T stages, positive lymph node status, and receipt of radiotherapy and chemotherapy were associated with worse overall and cancer-specific survival. RCS curves of each stage showed that the relationship between tumor size and prognosis was non-linear. Optimal cut-off points were identified based on the shape of RCS curves, suggesting new classifications of tumor size: 2.5 cm and 5 cm for Ta, 3 cm and 5 cm for T1, and 4 cm and 6 cm for T2.

**Conclusions:**

Incorporating tumor size into prognostic evaluations can enhance bladder cancer risk stratification. Further research is needed to validate these findings and improve personalized treatment strategies.

## Introduction

Rank as the ninth most prevalent cancer worldwide, bladder cancer (BC) maintains an incidence of 549,000 cases per year (1). The diagnosis and treatment of BC continue to be areas of active research due to the rapid advancements in molecular studies, pathology, and medical imaging (2–5). Over the past few decades, the tumor node metastasis (TNM) staging system established by the American Joint Committee on Cancer (AJCC) has consistently been the most valuable prognostic factor. Unlike many other tumors, the division of the T stage of BC relies primarily on the depth and extent of tumor invasion. However, researchers are still trying to enhance this staging system. For instance, the value of substage systems for non–muscle-invasive bladder cancer (NMIBC), categorized based on the extent of micrometric infiltration and invasion of the muscularis mucosae–vascular plexus, has been verified through retrospective studies (6, 7). Meanwhile, researchers have previously conducted a quantitative analysis of the depth of invasion in muscle-invasive bladder cancer (MIBC) to enhance the subclassification system (8). Nevertheless, as an essential feature of solid tumors, the significance of tumor size as a prognostic indicator and its potential incorporation into the staging system remains worthy of further investigation.

The European Organization for Research and Treatment of Cancer (EORTC) conducted the most renowned study on tumor size, demonstrating the significant predictive value of tumor size (>3 cm or ≤3 cm) in their prediction model for recurrence and progression in patients with Ta-T1 NMIBC (9). Tumor size is now recognized as a prognostic factor in risk stratification according to the European Association of Urology (EAU) Guideline and the National Comprehensive Cancer Network (NCCN) Clinical Practice Guideline, with a stratification threshold of 3 cm (10, 11). Studies have demonstrated the effectiveness of this approach and proposed further improvements in the stratification of tumor size (12, 13). However, the role of tumor size in MIBC, whether it is an independent prognostic factor in NMIBC, and its relationship with endoscopic resectability remains uncertain (14). Consequently, the application of tumor size is still very limited. Furthermore, tumor size has mainly been studied as categorical variables with artificially divided thresholds in exited studies, with tumor recurrence and progression being the commonly discussed outcomes. Given this background, we examined the correlation between tumor size and prognosis using the Surveillance, Epidemiology, and End Results (SEER) database. We employed restricted cubic splines (RCS) to visualize the non-linear relationship between tumor size and survival outcome, eventually determining the optimal cut-off value for categorizing size groups. Furthermore, we separately evaluated different size groups in NMIBC and MIBC to verify their prognostic significance.

## Materials and methods

### Study population

The SEER database, founded by the National Cancer Institute of the United States, is the largest cancer patient database, which encompasses survival information, tumor characteristics, treatment data, and demographic details for approximately 28% of the American population. Patients diagnosed with BC between 2004 and 2015 were selected from the SEER database. The inclusion criteria were as follows: (1) patients with bladder as the primary site of disease (coded as C670–679) and no distant metastases; (2) patients who underwent surgical intervention; (3) patients diagnosed with positive histological diagnosis. Patients with missing oncological information regarding tumor size, TNM stages, and grade were excluded from our study.

### Definition of variables

Demographic covariates included the year of diagnosis (2004–2009, 2009–2015), age group (≤60 years, 60–70 years, 70–80 years, >80 years), sex (male, female) and race (white, others). Tumor characteristics included tumor size, tumor grade (low grade, G1–G2; high grade, G3–G4), histology subtype (transitional cell carcinoma, squamous cell carcinoma, adenocarcinoma, neuroendocrine carcinoma, and others), T stage (Ta, Tis, T1, T2, T3, T4), and N stage (N0, N1, N2, N3). The TNM staging was based on the 6th version of the American Joint Committee on Cancer. Furthermore, the implementation of radiotherapy (yes, no/unknown) and chemotherapy (yes, no/unknown) was also considered.

### Statistical analyses

The categorical variables were reported as count (percentage), while tumor size was reported as a continuous variable using mean value (standard deviation) and median value (interquartile range). OS and CSS were calculated separately using the Kaplan-Meier method. Cox proportional hazard models were employed to estimate hazard ratios (HR) and corresponding 95% confidence intervals (95% CI). Firstly, we constructed univariate Cox models to identify significant variables. Subsequently, multivariate Cox regression models were employed, incorporating the significant variables. The Schoenfeld residuals test was used to verify the proportional hazards assumption, and the variance inflation factor (VIF) was employed to assess multicollinearity. After that, we used the RCS model to observe the association between tumor size and survival. The RCS curves were plotted separately for NMIBC, MIBC, each T stage, and each histology subtype. In addition, we divided the study population into different subgroups based on tumor size and plotted KM curves to investigate the prognostic value of these subgroups. The cutoff values for subgroup division were determined based on the shape of the RCS curves.

R version 4.2.2 (R Foundation for Statistical Computing) was utilized to conduct all statistical tests.

## Results

**Figure 1:**
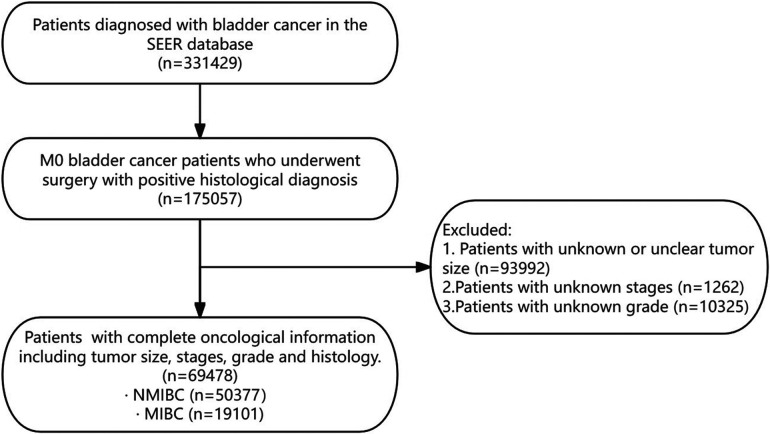
Flowchart of patient selection. SEER, surveillance, epidemiology, and end Results database; NMIBC, non-muscle invasive bladder cancer; MIBC, muscle-invasive bladder cancer.

A total of 69,478 patients were included in the study. The flowchart of patient selection is shown in Figure 1, and the baseline characteristics are shown in Table 1. The majority of patients were diagnosed between 2010 and 2015 (51.6%), aged 70–80 (30.2%), male (75.3%), of white race (89.7%), with high-grade tumors (57.3%) and transitional cell carcinoma (96.3%), at Ta stage (45.2%) and N0 stage (95.0%), and did not receive radiation therapy (95.6%) or chemotherapy (35.03%). The mean tumor size was 35.49 mm, with a standard deviation of 36.32 mm. The median size was 30 mm, with an interquartile range of 20–50.

**Table 1:** Baseline characteristics of patients with BC.

	Group	Number (*N* = 69,478)	Percentage (%)
Year of diagnosis	2004–2009	33,647	48.4
	2010–2015	35,831	51.6
Age	≤60	12,581	18.1
	60–70	18,357	26.4
	70–80	20,948	30.2
	>80	17,592	25.3
Sex	Male	52,331	75.3
	Female	17,147	24.7
Race	White	62,355	89.7
	Others	7,123	10.3
Grade	Low(G1–G2)	29,664	42.7
	High(G3–G4)	39,814	57.3
Histology	Transitional	66,880	96.3
	Squamous	1,244	1.8
	Adenocarcinoma	723	1.0
	Neuroendocrine	489	0.7
	Others	142	0.2
T stage	Ta	31,397	45.2
	Tis	1,835	2.6
	T1	17,145	24.7
	T2	11,277	16.2
	T3	5,110	7.4
	T4	2,714	3.9
N stage	N0	65,988	95.0
	N1	1,778	2.6
	N2	1,647	2.4
	N3	65	0.1
Radiation	No/Unknown	66,198	95.3
	Yes	3,280	4.7
Chemotherapy	No/Unknown	52,265	75.2
	Yes	17,213	24.8
Tumor size (mm)	Mean (SD)	35.49 (36.32)	—
	Median (IQR)	30.00 (20.00–50.00)	—

SD, standard deviation; IQR, interquartile range.

Cox regression models for OS and CSS, both univariable and multivariable, were constructed to calculate the hazard ratio of each variable (Tables 2, 3). Univariable analysis revealed that more recent years of diagnosis, older age, male, white people, high pathologic grade, non-transitional cell carcinoma, advanced T stages, positive lymph node status, and receipt of radiotherapy and chemotherapy were related to worse OS (all HR > 1, all *P* < 0.05). Additionally, tumor size was analyzed as a continuous variable and demonstrated an association with poorer OS (HR = 1.02, 95% CI 1.019–1.022, *P* < 0.001). When these variables (year of diagnosis, age, sex, race, grade, histology, T stage, N stage, radiotherapy, chemotherapy, `and tumor size) were included in the multivariable-adjusted Cox model, tumor size was identified as an independent risk factor of OS (HR = 1.013, 95% CI 1.012–1.015, *P* < 0.001). Comparable findings were observed in terms of CSS when tumor size was included in the univariable model (HR = 1.028, 95% CI 1.027–1.03, *P* < 0.001) and multivariable model (HR = 1.017, 95% CI 1.015–1.019, *P* < 0.001).

**Table 2:** Univariable and multivariable Cox regression analysis for OS in patients with BC.

Characteristics	Group			Univariate analysis		Multivariate analysis	
		HR	95% CI	P	HR	95% CI	P
Year of diagnosis	2004–2009	ref			ref		
	2010–2015	1.046	1.023–1.068	<0.001	1.01	0.988–1.033	0.358
Age	<60	ref			ref		
	60–70	1.674	1.607–1.743	<0.001	1.65	1.584–1.719	<0.001
	70–80	2.969	2.858–3.085	<0.001	2.945	2.835–3.06	<0.001
	>80	5.949	5.728–6.179	<0.001	5.877	5.656–6.107	<0.001
Sex	Female	ref			ref		
	Male	0.975	0.952–0.998	0.031	1.077	1.052–1.103	<0.001
Race	Others	ref			ref		
	White	1.048	1.013–1.085	0.007	1.053	1.018–1.09	0.003
Grade	Low(G1–G2)	ref			ref		
	High(G3–G4)	1.972	1.93–2.015	<0.001	1.165	1.134–1.197	<0.001
Histology	Transitional	ref			ref		
	Non-Transitional	1.93	1.842–2.022	<0.001	1.313	1.251–1.378	<0.001
T stage	Ta	0.648	0.631–0.665	<0.001	0.76	0.738–0.783	<0.001
	Tis	0.789	0.737–0.844	<0.001	0.861	0.804–0.922	<0.001
	T1	ref			ref		
	T2	1.825	1.771–1.88	<0.001	1.679	1.627–1.734	<0.001
	T3	2.077	2–2.156	<0.001	1.879	1.804–1.957	<0.001
	T4	3.281	3.135–3.434	<0.001	2.937	2.795–3.086	<0.001
N stage	N0	ref			ref		
	N1	2.634	2.498–2.777	<0.001	1.57	1.483–1.661	<0.001
	N2	3.4	3.222–3.588	<0.001	2.024	1.91–2.146	<0.001
	N3	4.606	3.552–5.973	<0.001	2.755	2.123–3.576	<0.001
Radiation	No/Unknown	ref			ref		
	Yes	2.884	2.774–2.999	<0.001	1.354	1.297–1.413	<0.001
Chemotherapy	No/Unknown	ref			ref		
	Yes	1.083	1.057–1.109	<0.001	0.837	0.815–0.859	<0.001
Tumor size(cm)		1.02	1.019–1.022	<0.001	1.013	1.012–1.015	<0.001

OS, overall survival; BC, bladder cancer; HR, hazard ratio; CI, confidence interval.

**Table 3:** Univariable and multivariable Cox regression analysis for CSS in patients With BC.

Characteristics	Group	Univariate analysis			Multivariate analysis		
		HR	95% CI	P	HR	95% CI	P
Year of diagnosis	2004–2009	ref			ref		
	2010–2015	1.095	1.061–1.131	<0.001	1.016	0.984–1.05	0.328
Age	<60	ref			ref		
	60–70	1.248	1.181–1.32	<0.001	1.224	1.157–1.294	<0.001
	70–80	1.742	1.653–1.836	<0.001	1.736	1.647–1.831	<0.001
	>80	3.156	2.997–3.323	<0.001	3.124	2.962–3.294	<0.001
Sex	Female	ref			ref		
	Male	0.814	0.786–0.843	<0.001	0.913	0.882–0.946	<0.001
Race	Others	ref			ref		
	White	0.853	0.812–0.896	<0.001	0.951	0.905–0.999	0.045
Grade	Low(G1–G2)	ref			ref		
	High(G3–G4)	4.521	4.338–4.711	<0.001	1.66	1.579–1.745	<0.001
Histology	Transitonal	ref			ref		
	Non-Transitional	3.105	2.932–3.288	<0.001	1.507	1.42–1.6	<0.001
T stage	Ta	0.347	0.33–0.366	<0.001	0.476	0.449–0.503	<0.001
	Tis	0.574	0.503–0.656	<0.001	0.7	0.613–0.8	<0.001
	T1	ref			ref		
	T2	2.983	2.856–3.116	<0.001	2.523	2.41–2.642	<0.001
	T3	3.914	3.723–4.115	<0.001	3.106	2.942–3.279	<0.001
	T4	6.337	5.984–6.71	<0.001	4.898	4.6–5.214	<0.001
N stage	N0	ref			ref		
	N1	4.898	4.609–5.206	<0.001	1.723	1.614–1.839	<0.001
	N2	6.364	5.993–6.757	<0.001	2.205	2.066–2.354	<0.001
	N3	8.806	6.688–11.595	<0.001	3.182	2.414–4.194	<0.001
Radiation	No/Unknown	ref			ref		
	Yes	4.136	3.939–4.343	<0.001	1.372	1.301–1.447	<0.001
Chemotherapy	No/Unknown	ref			ref		
	Yes	1.47	1.421–1.52	<0.001	0.819	0.789–0.851	<0.001
Tumor size(cm)		1.028	1.027–1.03	<0.001	1.017	1.015–1.019	<0.001

CSS, cancer-specific survival; BC, bladder cancer; HR, hazard ratio; CI, confidence interval.

**Figure 2:**
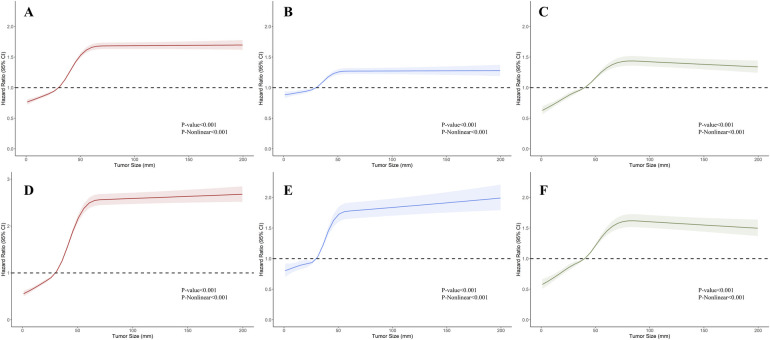
Restricted cubic splines showing the association between tumor size and survival in bladder cancer (BC). **(A–C)** Association between overall survival (OS) and tumor size in BC, non-muscle invasive bladder cancer (NMIBC), and muscle-invasive bladder cancer (MIBC). **(D–F)** Association between cancer-specific survival (CSS) and tumor size in BC, NMIBC, and MIBC.

**Figure 3:**
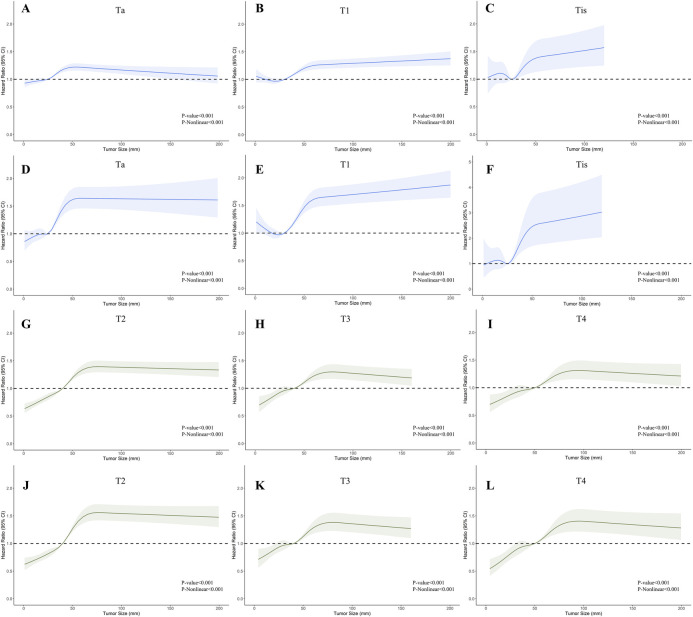
Restricted cubic splines showing the association between tumor size and survival across different T stages of bladder cancer (BC). **(A–C)**; **(G–I)** Association between overall survival (OS) and tumor size in stages Ta, T1, Tis, T2, T3, and T4. **(D–F)**; **(J–L)** Association between cancer-specific survival (CSS) and tumor size in stages Ta, T1, Tis, T2, T3, and T4.

To visualize the impact of tumor size on prognosis, we employed RCS models based on Cox proportional hazards models, using 5 knots to illustrate the non-linear relationship. In the RCS models for the general population, the risk of OS and CSS exhibited a moderate growth for tumor sizes below 3 cm, followed by a rapid increase once tumor size exceeded 3 cm. Eventually, it plateaued after reaching 5 cm (Figures 2A,D). This trend diverged after stratification by muscle infiltration condition, with the moderate growth of HR in MIBC continuing until the tumor size reached 4 cm and then stabilized at around 6 cm (Figures 2C,F). We constructed RCS curves at various T stages to investigate this trend further. As shown in Figure 3, RCS curves across various stages exhibit a similar pattern, with a relatively gradual increase in risk when the tumor is small, followed by a more rapid escalation in the mid-range and eventual stabilization after reaching a specific size. This pattern is notably distinct in Ta, T1, and T2 tumors, while trends in Tis, T3, and T4 tumors are relatively ambiguous with relatively wide confidence intervals. We also investigated the relationship between histology subtypes and tumor size. Compared to transitional cell carcinoma, bladder tumors with variant histology showed a generally larger tumor size (Supplementary Table S1). In Supplementary Figure S1, while the overall trends are consistent, the RCS curves for different pathological types show distinct characteristics. Notably, the RCS curve for squamous cell carcinoma has high statistical significance; its slope is relatively gentle compared to the curve of transitional cell carcinoma, and the turning point is not very pronounced. Due to the limited sample size, the statistical significance of the adenocarcinoma and neuroendocrine carcinoma curves is relatively low. Furthermore, we utilized the HR value estimated by the RCS model, abscissa calculated by interpolation functions, along with the shapes of the curves, to determine the optimal value of the inflection points in Ta, T1, and T2. The inflection points were specified as 2.5 cm and 5 cm in Ta, 3 cm and 5 cm in T1, and 4 cm and 6 cm in T2, respectively.

**Figure 4:**
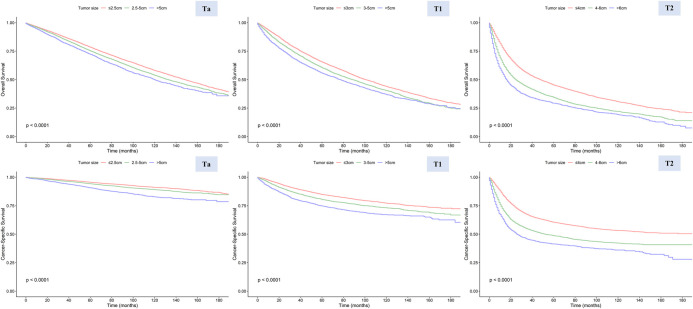
Overall survival (OS) and cancer-specific survival (CSS) in stages Ta, T1, and T2 patients stratified by different tumor sizes.

Based on cutoff values determined by RCS curve shapes, we divided patients into different subgroups, and the Kaplan-Meier method was utilized to evaluate the significance of these grouping methods (Figure 4). All KM curves exhibited significant differences between the groups, suggesting the efficacy of these classification methods. Both univariate and multivariate Cox regression analyses indicated that the size-based grouping method could more effectively discern survival risks (15, 16) (Supplementary Table S2). In the multivariate models of CSS, the stratification strategies performed well for patients in groups Ta (2.5–5.0 cm vs. <=2.5 cm: HR = 1.186, 95% CI 1.09–1.291, *P* < 0.001; >5.0 cm vs. <=2.5 cm: HR = 1.822, 95% CI 1.604–2.07), T1 (3.0–5.0 cm vs. <=3.0 cm: HR = 1.243, 95% CI 1.152–1.342, *P* < 0.001; >5.0 cm vs. <=3.0 cm: HR = 1.618, 95% CI 1.478–1.77), and T2 (4.0–6.0 cm vs. <=4.0 cm: HR = 1.442, 95% CI 1.354–1.535, *P* < 0.001; >6.0 cm vs. <=4.0 cm: HR = 2.049, 95% CI 1.895–2.215). In addition, we validated the grouping method using the SEER dataset for 2016–2017, with information provided in the Supplementary Materials (Supplementary Figure S2 and Supplementary Table S3).

## Discussion

In recent years, research efforts have been focused on establishing models for risk stratification and prognosis forecasting on BC, particularly NMIBC. The EORTC initially formulated a calculation system for NMIBC, drawing data from a series of randomized trials (17–21), revealing a significant correlation between tumor size, number of tumors, prior recurrence rate, and the likelihood of recurrence. Furthermore, T stage, the presence of cancer *in situ*, and grade were proved to be the most important prognosis factors for progression (9). However, the tumor size was artificially divided into <3 cm and ≥3 cm without differentiation among sub-stages or further exploration of the cut-off value. Another stratification model was developed by the Spanish Urological Club for Oncological Treatment, which segmented tumor sizes at 1 cm and 3 cm thresholds. Nevertheless, this parameter was not integrated into their final scoring model (22). The risk stratification standard established by the American Urological Association (AUA) also took 3 cm as the critical threshold of stratifying (23). In the meantime, the role of tumor size in MIBC undergoing radical cystectomy remains uncertain (14); only a limited number of studies have suggested that tumor size in pT2 is predictive of CSS or metastasis-free survival (8). Using the SEER database, our study meticulously examined the relationship between tumor size and prognosis in BC. By employing RCS, we graphically represented these associations, ultimately determining optimal cut-off values for categorizing tumor sizes within certain T stages, providing a reference for further improvement of the staging system or establishing prognosis models.

For NMIBC, this paper suggests some improvements to the existing classification criteria. First, 5 cm is considered a valuable cut-off point, except for 3 cm. This trend is particularly discernible on the RCS curve, where once the tumor size surpasses 5 cm, the impact of size escalation on HR significantly diminishes. Secondly, the 2.5 cm for Ta-stage tumors emerges as a more critical inflection point than 3 cm for T1-stage tumors, which may be attributed to the shallower invasion depth of Ta-stage tumors and their better prognosis. However, the rationality and practicality of this conclusion remain uncertain, given the difficulties in precisely measuring tumor size in patients receiving transurethral resection of bladder tumors (TURBT). In addition, the prognostic value of tumor size in Tis-stage tumors is lacking, which is evident because the lesion is confined to the mucosal layer. For MIBC, our study found that using 4 cm and 6 cm as classification criteria effectively supplements the current T2 tumor classification based on the depth of muscle layer invasion. Furthermore, the RCS curves for T3 and T4 tumors were relatively irregular; since the main factor affecting the prognosis of these tumors was their invasion into tissues surrounding the bladder, we did not include them in the subsequent analysis.

The variant histology (VH) of bladder cancer is also a noteworthy topic. In our research, non-transitional carcinoma demonstrated significantly larger tumor sizes and worse survival outcomes, with RCS curves exhibiting different characteristics. Researchers have focused on the characters and prognostic significance of histological subtypes (24, 25). As the most common VH of BC, squamous cell carcinoma was described as bulky and polypoid, filling the bladder cavity (26). In our study, squamous cell carcinoma exhibited the largest mean tumor size, with its RCS curves showing indistinct segmentation features. This may also reflect its overall more aggressive nature. A similar trend was observed in adenocarcinoma, while changes in the size of neuroendocrine carcinoma below 5 cm had less impact on survival. Thus, adopting a uniform management approach for non-transitional carcinomas is unwise; targeted research for reinterpretation is needed.

The main advantages of our study comprised the analysis of large sample sizes, the visualization of trends in continuous variables, and an in-depth exploration based on these findings. A limited number of studies have discussed the association between tumor size and prognosis. Tully et al. analyzed the impact of tumor size on oncological outcomes in 1,116 patients with high-grade NMIBC and demonstrated that patients with tumors larger than 3 cm have worse OS and CSS (13). Gofrit et al. and Lee et al. developed and validated the classification of patients with tumors smaller than 1 cm as “very low risk” (12, 27). However, it is essential to note that an efficient method is absent for accurately measuring such scales, and a larger threshold appears more reasonable for both imaging and visual observation. Meanwhile, these studies involved small sample sizes and either employed an artificially defined classification size or validated a previous classification, resulting in limited practical value and generalizability. Nevertheless, our study's combination of RCS, KM analysis, and COX regression scientifically reflect the relationship, and the large population enhances its applicability in clinical practice. The preliminary assessment of tumor size based on imaging and cystoscopy is more timely than that based on postoperative pathology. Additionally, tumor size classification can further aid in prognostic evaluation in postoperative patients combined with current TNM staging.

Our study has some limitations. First, as a retrospective study, the data quality is constrained by uncontrollable interventions and inherent biases, such as inaccuracies in tumor size measurement. Additionally, the study cohort primarily consists of individuals from the United States, raising concerns about the generalizability of the results. Validation across diverse populations may be necessary to ensure that the conclusions drawn from this study are broadly applicable. The accurate measurement of tumor size is also a challenge. Since TURBT is the primary procedure for NMIBC, the measurement of tumor size primarily relies on imaging examination or visual assessment using an endoscope, which undoubtedly lacks precision. The development of measurement techniques, such as imaging omics and endoscopic precision measurement, may represent a valuable research direction. Additionally, the SEER database only provides data on chemotherapy and radiotherapy, lacking information on essential treatments of BC, such as intravesical chemotherapy, BCG therapy, and immunotherapy. These treatments' application efficacy and impact on survival outcomes cannot be further analyzed (28). Due to a similar issue, only OS and CSS information are provided. As the vital outcome indicators, progression-free and recurrence-free survival could not be assessed.

In conclusion, this study further explores the correlation between BC tumor size and prognosis, introducing novel stratification criteria. It emphasizes the significance of integrating tumor size assessments into prognostic evaluations to improve risk stratification and patient management strategies. Prospective studies are needed to validate these new classifications' value further. In addition, further studies are warranted to personalize treatment for different pathological subtypes of BC and explore the relationship between tumor size and prognosis from molecular biology's perspective (29).

## Data Availability

Publicly available datasets were analyzed in this study. This data can be found here: https://seer.cancer.gov/.

## Supplementary material

Supplementary Figure S1 Restricted cubic splines showing the association between tumor size and survival across different histological subtypes. (A) OS across different histological subtypes (B) CSS across different histological subtypes.

Supplementary Figure S2 Validation of the efficacy of the classification scheme in patients from 2016 to 2017 sourced from the SEER database. (A-C) OS in stages Ta, T1, and T2 (D-F) CSS in stages Ta, T1, and T2.

## References

[1] Antoni S Ferlay J Soerjomataram I Znaor A Jemal A Bray F. Bladder cancer incidence and mortality: a global overview and recent trends. Eur Urol. (2017) 71(1):96–108. 10.1016/j.eururo.2016.06.010 27370177

[2] Compérat E Amin MB Cathomas R Choudhury A De Santis M Kamat A Current best practice for bladder cancer: a narrative review of diagnostics and treatments. The Lancet. (2022) 400(10364):1712–21. 10.1016/S0140-6736(22)01188-6 36174585

[3] Panebianco V Narumi Y Altun E Bochner BH Efstathiou JA Hafeez S Multiparametric magnetic resonance imaging for bladder cancer: development of VI-RADS (vesical imaging-reporting and data system). Eur Urol. (2018) 74(3):294–306. 10.1016/j.eururo.2018.04.029 29755006 PMC6690492

[4] Humayun-Zakaria N Ward DG Arnold R Bryan RT. Trends in urine biomarker discovery for urothelial bladder cancer: DNA, RNA, or protein? Transl Androl Urol. (2021) 10(6):2787–808. 10.21037/tau-20-1327 34295762 PMC8261432

[5] Kamoun A de Reyniès A Allory Y Sjödahl G Robertson AG Seiler R A consensus molecular classification of muscle-invasive bladder cancer. Eur Urol. (2020) 77(4):420–33. 10.1016/j.eururo.2019.09.006 31563503 PMC7690647

[6] van Rhijn BWG van der Kwast TH Alkhateeb SS Fleshner NE van Leenders GJLH Bostrom PJ A new and highly prognostic system to discern T1 bladder cancer substage. Eur Urol. (2012) 61(2):378–84. 10.1016/j.eururo.2011.10.026 22036775

[7] Colombo R Hurle R Moschini M Freschi M Colombo P Colecchia M Feasibility and clinical roles of different substaging systems at first and second transurethral resection in patients with T1 high-grade bladder cancer. Eur Urol Focus. (2018) 4(1):87–93. 10.1016/j.euf.2016.06.004 28753746

[8] Cheng L Neumann RM Scherer BG Weaver AL Leibovich BC Nehra A Tumor size predicts the survival of patients with pathologic stage T2 bladder carcinoma: a critical evaluation of the depth of muscle invasion. Cancer. (1999) 85(12):2638–47. 10.1002/(sici)1097-0142(19990615)85:12<2638::aid-cncr21>3.0.co;2-v 10375113

[9] Sylvester RJ van der Meijden APM Oosterlinck W Witjes JA Bouffioux C Denis L Predicting recurrence and progression in individual patients with stage Ta, T1 bladder cancer using EORTC risk tables: a combined analysis of 2596 patients from seven EORTC trials. Eur Urol. (2006) 49(3):466–77. 10.1016/j.eururo.2005.12.031 16442208

[10] Babjuk M Burger M Capoun O Cohen D Compérat EM Dominguez Escrig JL European association of urology guidelines on non-muscle-invasive bladder cancer (Ta, T1, and carcinoma in situ). Eur Urol. (2022) 81(1):75–94. 10.1016/j.eururo.2021.08.010 34511303

[11] Flaig TW Spiess PE Agarwal N Bangs R Boorjian SA Buyyounouski MK Bladder cancer, version 3.2020, NCCN clinical practice guidelines in oncology. J Natl Compr Cancer Netw. (2020) 18(3):329–54. 10.6004/jnccn.2020.0011 32135513

[12] Lee A Lee HJ Huang HH Ho H Chen K. Low-risk non-muscle-invasive bladder cancer: further prognostic stratification into the “very-low-risk” group based on tumor size. Int J Urol Off J Jpn Urol Assoc. (2019) 26(4):481–6. 10.1111/iju.13913 30834632

[13] Tully KH Moschini M von Rundstedt F-CE Aziz A Kluth LA Necchi A Impact of tumor size on the oncological outcome of high-grade nonmuscle invasive bladder cancer—examining the utility of classifying Ta bladder cancer based on size. Urol Oncol. (2020) 38(11):851.e19–e25. 10.1016/j.urolonc.2020.06.034 32739227

[14] Hensley PJ Panebianco V Pietzak E Kutikov A Vikram R Galsky MD Contemporary staging for muscle-invasive bladder cancer: accuracy and limitations. Eur Urol Oncol. (2022) 5(4):403–11. 10.1016/j.euo.2022.04.008 35581143

[15] Chen X Xu R He D Zhang Y Chen H Zhu Y CD8+ T effector and immune checkpoint signatures predict prognosis and responsiveness to immunotherapy in bladder cancer. Oncogene. (2021) 40(43):6223–34. 10.1038/s41388-021-02019-6 34552192

[16] Chen X Chen H Yao H Zhao K Zhang Y He D Turning up the heat on non-immunoreactive tumors: pyroptosis influences the tumor immune microenvironment in bladder cancer. Oncogene. (2021) 40(45):6381–93. 10.1038/s41388-021-02024-9 34588621

[17] Bouffioux CH Denis L Oosterlinck W Viggiano G Vergison B Keuppens F Adjuvant chemotherapy of recurrent superficial transitional cell carcinoma: results of a European organization for research on treatment of cancer randomized trial comparing intravesical instillation of thiotepa, doxorubicin and cisplatin. J Urol. (1992) 148(2):297–301. 10.1016/S0022-5347(17)36577-1 1635122

[18] Newling DW Robinson MR Smith PH Byar D Lockwood R Stevens I Tryptophan metabolites, pyridoxine (vitamin B6) and their influence on the recurrence rate of superficial bladder cancer. Eur Urol. (1995) 27(2):110–6. 10.1159/000475139 7744151

[19] Kurth K Tunn U Ay R Schröder FH Pavone-Macaluso M Debruyne F Adjuvant chemotherapy for superficial transitional cell bladder carcinoma: long-term results of a European organization for research and treatment of cancer randomized trial comparing doxorubicin, ethoglucid and transurethral resection alone. J Urol. (1997) 158(2):378–84. 10.1016/s0022-5347(01)64484-7 9224307

[20] Bouffioux C Kurth KH Bono A Oosterlinck W Kruger CB De Pauw M Intravesical adjuvant chemotherapy for superficial transitional cell bladder carcinoma: results of 2 European organization for research and treatment of cancer randomized trials with mitomycin C and doxorubicin comparing early versus delayed instillations and short-term versus long-term treatment. J Urol. (1995) 153(3 Pt 2):934–41. 7853578

[21] Oosterlinck W Kurth KH Schröder F Bultinck J Hammond B Sylvester R. A prospective European organization for research and treatment of cancer genitourinary group randomized trial comparing transurethral resection followed by a single intravesical instillation of epirubicin or water in single stage Ta, T1 papillary carcinoma of the bladder. J Urol. (1993) 149(4):749–52. 10.1016/s0022-5347(17)36198-0 8455236

[22] Fernandez-Gomez J Madero R Solsona E Unda M Martinez-Piñeiro L Gonzalez M Predicting nonmuscle invasive bladder cancer recurrence and progression in patients treated with bacillus Calmette-Guerin: the CUETO scoring model. J Urol. (2009) 182(5):2195–203. 10.1016/j.juro.2009.07.016 19758621

[23] Chang SS Boorjian SA Chou R Clark PE Daneshmand S Konety BR Diagnosis and treatment of non-muscle invasive bladder cancer: AUA/SUO guideline. J Urol. (2016) 196(4):1021–9. 10.1016/j.juro.2016.06.049 27317986

[24] Claps F Biasatti A Di Gianfrancesco L Ongaro L Giannarini G Pavan N The prognostic significance of histological subtypes in patients with muscle-invasive bladder cancer: an overview of the current literature. J Clin Med. (2024) 13(15):4349. 10.3390/jcm13154349 39124615 PMC11313590

[25] Mori K Abufaraj M Mostafaei H Quhal F Karakiewicz PI Briganti A A systematic review and meta-analysis of variant histology in urothelial carcinoma of the bladder treated with radical cystectomy. J Urol. (2020) 204(6):1129–40. 10.1097/JU.0000000000001305 32716694

[26] Gellert LL Warrick J Al-Ahmadie HA. Urothelial carcinoma with squamous differentiation–the pathologists' perspective. Urol Oncol. (2015) 33(10):437–43. 10.1016/j.urolonc.2015.07.018 26321057

[27] Gofrit ON Pode D Pizov G Duvdevani M Landau EH Hidas G “Very-low-risk” Bladder Tumours—A New Entity? BJU Int. (2018) 121(4):627–31. 10.1111/bju.14108 29274202

[28] Claps F Pavan N Ongaro L Tierno D Grassi G Trombetta C BCG-unresponsive non-muscle-invasive bladder cancer: current treatment landscape and novel emerging molecular targets. Int J Mol Sci. (2023) 24(16):12596. 10.3390/ijms241612596 37628785 PMC10454200

[29] Chen X Chen H He D Cheng Y Zhu Y Xiao M Analysis of tumor microenvironment characteristics in bladder cancer: implications for immune checkpoint inhibitor therapy. Front Immunol. (2021) 12:672158. 10.3389/fimmu.2021.672158 33936117 PMC8082152

