# Supplementary material for: Association between tumor size and prognosis in bladder cancer: novel classifications and insights from a SEER database analysis
Source: Front Surg. 2024 Nov 25;11:1489832. doi: 10.3389/fsurg.2024.1489832 (PMC11625752; doi:10.3389/fsurg.2024.1489832)
Supplement: Supplementary file 1 [file Table1.docx]

**Supplementary Table 1.** Tumor Size in Histology Subtypes

| Tumor size(mm) | Transitional Cell Carcinoma | Squamous Cell Carcinoma | Adenocarcinoma | Neuroendocrine  Carcinoma | Others |
| --- | --- | --- | --- | --- | --- |
| Mean (SD) | 34.93 (35.56) | 56.25 (63.45) | 41.43 (26.85) | 47.24 (36.67) | 45.60 (41.99) |
| Median (IQR) | 30.00 (20.00-50.00) | 50.00(30.00-70.00) | 43.00(24.50-50.00) | 43.00(30.00-56.00) | 40.50(25.00-51.00) |

SD, Standard Deviation; IQR, Interquartile Range
