# Supplementary material for: Association between tumor size and prognosis in bladder cancer: novel classifications and insights from a SEER database analysis
Source: Front Surg. 2024 Nov 25;11:1489832. doi: 10.3389/fsurg.2024.1489832 (PMC11625752; doi:10.3389/fsurg.2024.1489832)
Supplement: Supplementary file 2 [file Table2.docx]

**Supplementary Table 1.** Cox Regression Analysis for Survival Stratified by Tumor Size

|  |  | Hazard Ratio (95%CI) | | | |
| --- | --- | --- | --- | --- | --- |
|  |  | OS | | CSS | |
|  | Size (cm) | Univariate | Adjusted | Univariate | Adjusted |
| Ta | <=2.5 | ref | ref | ref | ref |
|  | 2.5-5.0 | 1.134(1.094-1.176) | 1.124(1.083-1.167) | 1.253(1.151-1.363) | 1.186(1.09-1.291) |
|  | >5.0 | 1.278(1.2-1.362) | 1.265(1.184-1.351) | 1.995(1.758-2.264) | 1.822(1.604-2.07) |
| T1 | <=3.0 | ref | ref | ref | ref |
|  | 3.0-5.0 | 1.154(1.103-1.208) | 1.12(1.07-1.173) | 1.287(1.193-1.389) | 1.243(1.152-1.342) |
|  | >5.0 | 1.297(1.224-1.374) | 1.274(1.203-1.35) | 1.710(1.563-1.87) | 1.618(1.478-1.77) |
| T2 | <=4.0 | ref | ref | ref | ref |
|  | 4.0-6.0 | 1.382(1.315-1.452) | 1.342(1.277-1.41) | 1.497(1.407-1.594) | 1.442(1.354-1.535) |
|  | >6.0 | 1.675(1.572-1.785) | 1.806(1.694-1.925) | 1.954(1.808-2.112) | 2.049(1.895-2.215) |

OS: Overall Survival; CSS: Cancer-Specific Survival; BC: Bladder Cancer; HR: Hazard ratio; CI: confidence interval.
