# Supplementary material for: Association between tumor size and prognosis in bladder cancer: novel classifications and insights from a SEER database analysis
Source: Front Surg. 2024 Nov 25;11:1489832. doi: 10.3389/fsurg.2024.1489832 (PMC11625752; doi:10.3389/fsurg.2024.1489832)
Supplement: Supplementary file 3 [file Table3.docx]

**Supplementary Table 2.** Cox Regression Analysis for Survival Stratified by Tumor Size ( 2016-2017 Cohort)

|  |  | Hazard Ratio (95%CI) | | | |
| --- | --- | --- | --- | --- | --- |
|  |  | OS | | CSS | |
|  | Size (cm) | Univariate | Adjusted | Univariate | Adjusted |
| Ta | <=2.5 | ref | ref | ref | ref |
|  | 2.5-5.0 | 1.198(1.050-1.367) | 1.176(1.030-1.343) | 1.703(1.262-2.299) | 1.591(1.76-2.151) |
|  | >5.0 | 1.775(1.458-2.162) | 1.634(1.337-1.998) | 3.176(2.141-4.710) | 2.566(1.712-3.845) |
| T1 | <=3.0 | ref | ref | ref | ref |
|  | 3.0-5.0 | 1.208(1.042-1.401) | 1.232(1.062-1.429) | 1.491(1.201-1.851) | 1.483(1.194-1.842) |
|  | >5.0 | 1.588(1.349-1.869) | 1.598(1.354-1.885) | 2.189(1.7741-2.751) | 2.113(1.674-2.669) |
| T2 | <=4.0 | ref | ref | ref | ref |
|  | 4.0-6.0 | 1.376(1.207-1.568) | 1.327(1.163-1.513) | 1.379(1.184-1.606) | 1.325(1.137-1.545) |
|  | >6.0 | 1.767(1.499-2.083) | 1.788(1.516-2.109) | 1.973(1.640-2.374) | 1.982(1.6646-2.386) |

OS: Overall Survival; CSS: Cancer-Specific Survival; BC: Bladder Cancer; HR: Hazard ratio; CI: confidence interval.
