# Supplementary figures and images for: Association between tumor size and prognosis in bladder cancer: novel classifications and insights from a SEER database analysis
Source: Front Surg. 2024 Nov 25;11:1489832. doi: 10.3389/fsurg.2024.1489832 (PMC11625752; doi:10.3389/fsurg.2024.1489832)

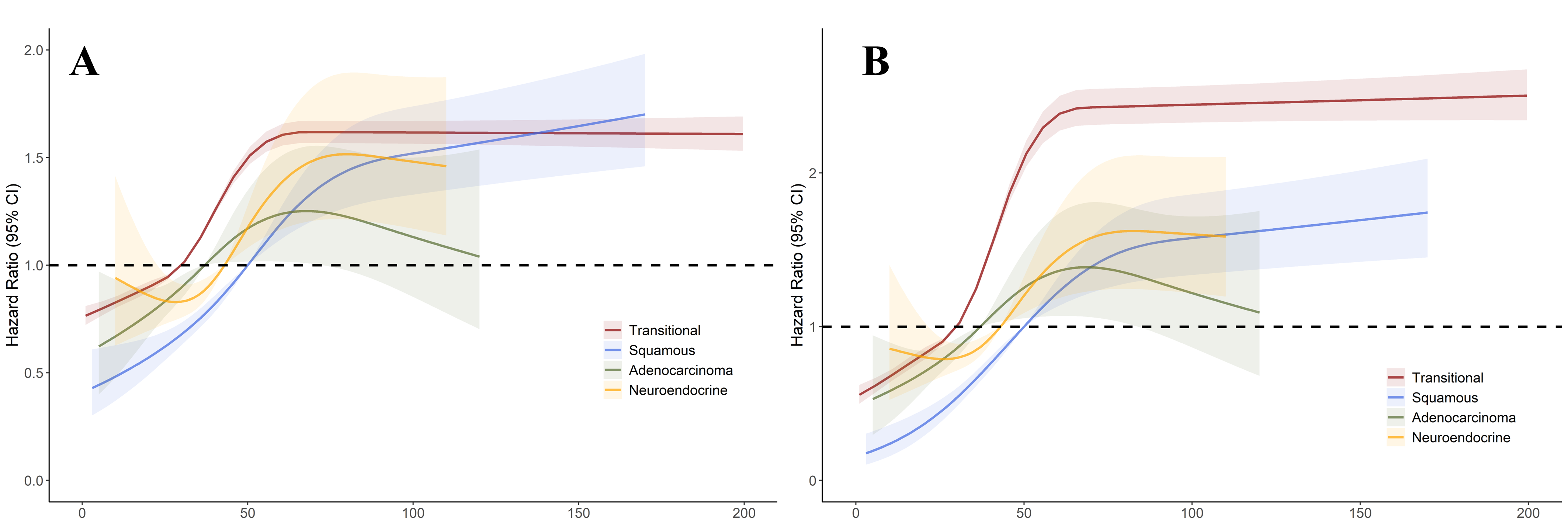

Supplement: Supplementary Figure S1 — Restricted cubic splines showing the association between tumor size and survival across different histological subtypes. (A) OS across different histological subtypes (B) CSS across different histological subtypes. [file Image1.tif]

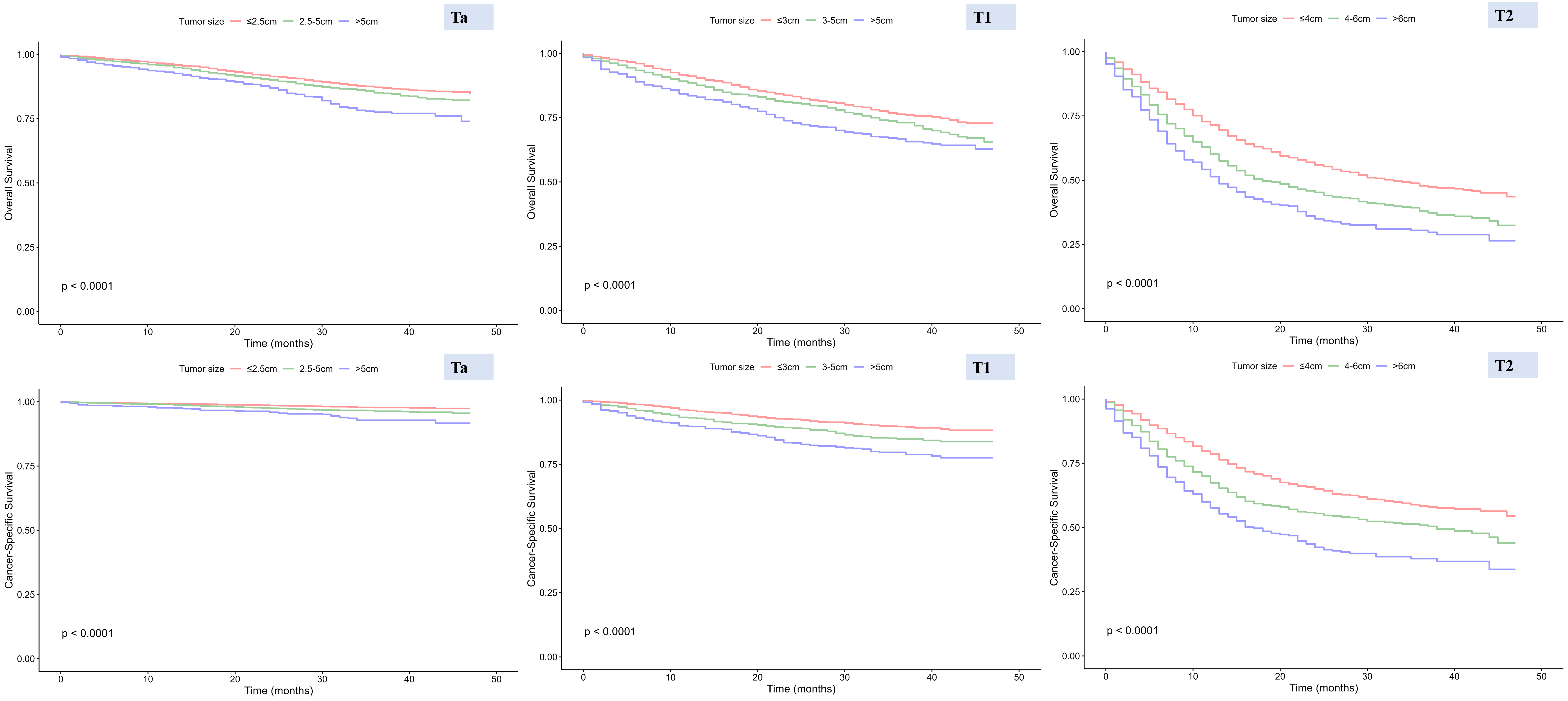

Supplement: Supplementary Figure S2 — Validation of the efficacy of the classification scheme in patients from 2016 to 2017 sourced from the SEER database. (A-C) OS in stages Ta, T1, and T2 (D-F) CSS in stages Ta, T1, and T2. [file Image2.tif]
